# Effects of Captivity on the Morphology of the Insertion Sites of the Palmar Radiocarpal Ligaments in Hominoid Primates

**DOI:** 10.3390/ani11071856

**Published:** 2021-06-22

**Authors:** Aroa Casado, Yasmina Avià, Miquel Llorente, David Riba, Juan Francisco Pastor, Josep Maria Potau

**Affiliations:** 1 Unit of Human Anatomy and Embryology, University of Barcelona, 08036 Barcelona, Spain; aroa.casado@ub.edu; 2 Faculty of Geography and History, Institut d’Arqueologia de la Universitat de Barcelona, University of Barcelona, 08001 Barcelona, Spain; y.avia@ub.edu; 3 Department of Evolutionary Biology, Ecology and Environmental Sciences, University of Barcelona, 08028 Barcelona, Spain; 4 Department of Psychology, Serra Húnter Fellow, University of Girona, 17004 Girona, Spain; miguel.llorente@udg.edu; 5 Department of History and History of Art, University of Girona, 17004 Girona, Spain; david.ribacano@udg.edu; 6 Department of Anatomy and Radiology, University of Valladolid, 47005 Valladolid, Spain; juanpas@med.uva.es

**Keywords:** wrist anatomy, hominoid primates, captivity

## Abstract

**Simple Summary:**

In this manuscript, we report the results of our 3D geometric morphometric analyses of the distal radial epiphysis in wild and captive gorillas, chimpanzees, and orangutans. We have identified significant differences in the insertion sites of the palmar radiocarpal ligaments between the wild and captive specimens of each species that are likely related to the locomotor behaviors developed in captivity. We believe that our study deals with a subject of great social impact in today’s world: the well-being of animals living in captivity, especially hominoid primates. Our findings provide novel information on the effect of captivity on the anatomy and locomotor behavior of hominoid primates. We trust that this information can be a basis for improving the artificial spaces where these captive primates live by increasing their available space and providing structures that more closely simulate their natural environment.

**Abstract:**

The environmental conditions of captive hominoid primates can lead to modifications in several aspects of their behavior, including locomotion, which can then alter the morphological characteristics of certain anatomical regions, such as the knee or wrist. We have performed tridimensional geometric morphometrics (3D GM) analyses of the distal radial epiphysis in wild and captive gorillas, chimpanzees, and orangutans. Our objective was to study the morphology of the insertion sites of the palmar radiocarpal ligaments, since the anatomical characteristics of these insertion sites are closely related to the different types of locomotion of these hominoid primates. We have identified significant differences between the wild and captive specimens that are likely related to their different types of locomotion. Our results indicate that the habitat conditions of captive hominoid primates may cause them to modify their locomotor behavior, leading to a greater use of certain movements in captivity than in the wild and resulting in the anatomical changes we have observed. We suggest that creating more natural environments in zoological facilities could reduce the impact of these differences and also increase the well-being of primates raised in captive environments.

## 1. Introduction

Osteological studies of animals are generally carried out with specimens from reference collections, such as research centers and museums. Many of these specimens come from zoos, which provide animals that have died from different causes. However, some investigators believe that osteological studies are more reliable when conducted using specimens of wild animals, since there may be differences in the anatomical characteristics of bones from wild and captive animals [1,2]. Some groups of animals, such as hominoid primates, are highly susceptible to the development of physical or behavioral alterations under captive conditions [3]. The musculoskeletal system is directly related to locomotion, which is one of the physiological activities with greater differences between wild and captive animals, as can be observed in different species of primates and carnivores [4,5,6,7], making it an especially interesting area of study.

In the field of primatology, several studies have explored the differences between some parameters related to the habitats of wild and captive primates [8,9,10]. For example, the natural habitats of wild hominoid primates are characterized by the presence of trees or geological elements with a great variation in height, inclination, hardness, and, especially, space [11]. In contrast, the habitats of captive hominoid primates are characterized by small enclosures that have virtually non-existent or highly limited substrate complexity compared to the substrate of their natural habitat [12]. Moreover, in captivity the size of the space is less important than its complexity [13,14,15,16,17,18], which can be increased by providing structures that simulate the natural environment of the primates so that they can use them as they would under natural conditions [12]. However, some studies show naturalistic environments are important but not essential to generate adequate living conditions for their inhabitants [19]. Other authors stressed the importance of developing species-typical and natural behaviors [20], also called ethnological needs [21,22]. To achieve these behaviors, animals require complex environments and stimuli [23]. Physical, social, sensory, and cognitive stimulation through environmental enrichment may increase behavioral opportunities and enhance welfare, becoming a key element in captive animal care and management [24,25].

Studies quantifying the time spent by captive hominoid primates in occupying certain spaces have found that, in naturalized environments with trees and elevated structures, orangutans prefer to spend most of their time in elevated structures [16], while gorillas prefer to stay near enclosure buildings or on their upper floors, on large trees, and on rocks [12,17,26] and chimpanzees generally prefer small spaces that are elevated and away from the public [12,15]. Captive gorillas also tend to use vertical structures more frequently than wild gorillas since the structures in captivity are generally made of artificial material that is less likely to break than the structures found in the wild [27]. For the same reason, the suspensory locomotion of captive orangutans is generally faster and less cautious than that of wild orangutans [7]. In addition to the behavioral changes that can occur in captive primates due to the physical characteristics and complexity of their habitats, other modifications of their routines such as changes in the composition of their groups or modifications in their home can also significantly alter their development [12]. Several investigators have argued that the habitats of captive primates generate atypical cognitive [28] and locomotor behaviors [2,7,29] as well as nutrition problems that can lead to obesity [30,31], ultimately impacting their growth, physiology, and behavior [32,33].

In recent years, a number of studies have explored the anatomical differences between wild and captive animals of different species. Many have focused on differences in the cranial region [10,34], while others have examined specific regions of postcranial anatomy [2,35,36]. Although studies on the effects of captivity on the postcranial skeleton are scarce [2,4,5,6,35,36], most indicate that different regions of the postcranial skeleton respond in different ways to habitat conditions [2,9]; some bone structures show a greater tendency to present anatomical differences between wild and captive primates, while others remain morphologically stable [35,36]. For example, no significant differences in scapula morphology have been identified between wild and captive hominoid primates [35], nor have differences in the length of different anatomical regions (total body, upper arm, lower arm, hand, upper leg, lower leg, tail, foot, head, and canine) been identified in vervet monkeys [36]. However, different species of primates have been shown to respond differently to captivity [12]. Significant morphological differences have been reported in some long bones between wild and captive gorillas [9] and in the wrist and knee between wild and captive chimpanzees [2].

**Figure 1:**
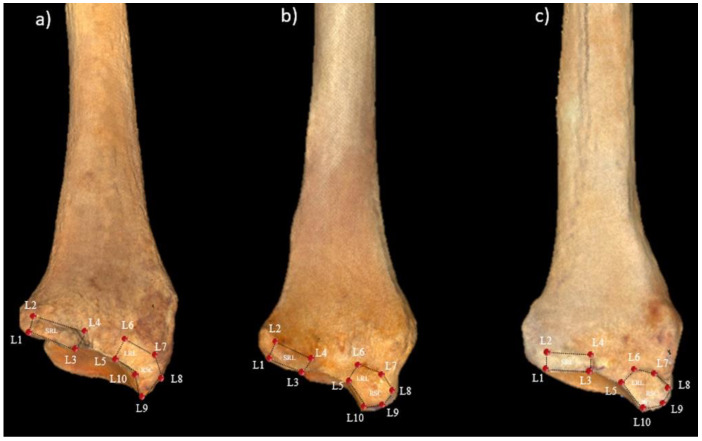
Distal radial epiphyses of (**a**) *Gorilla gorilla*, (**b**) *Pan troglodytes*, and (**c**) *Pongo pygmaeus*. In each of the distal radial epiphyses, the insertion sites of the palmar radiocarpal ligaments are shown with the locations of the landmarks.

In the wrist of chimpanzees, for example, the articular surfaces of the distal radius and distal ulna are larger in captive than in wild chimpanzees [2], likely because captive chimpanzees use knuckle-walking more than wild chimpanzees during their youth [2]. This may be due to differences between captive and wild environments, as captive environments generally have less three-dimensional complexity, less variation in heights and slopes, and more hard elements like artificial rocks and cement floors [2]. The palmar radiocarpal ligaments are the main stabilizing elements of the radiocarpal joint [37], and their insertion sites are located in the distal radial epiphysis [38] (Figure 1). The common insertion site of the radioscaphocapitate ligament (RSC) and the long radiolunate ligament (LRL), as well as that of the short radiolunate ligament (SRL), have previously been studied by our group in three species of hominoid primates (*Gorilla gorilla, Pan troglodytes* and *Pongo pygmaeus*). We found that the morphology of these two insertion sites was related to the type of locomotion used by each of the species. The insertion sites in the more arboreal species, like *Pongo pygmaeus,* are larger, with a palmar orientation of the SRL ligament insertion site, while those of the knuckle-walkers (*Gorilla gorilla*) are smaller, with an ulnopalmar orientation of the SRL ligament insertion site, presenting *Pan troglodytes* an intermediate position [38]. This relationship between the morphology of a ligament insertion site and type of locomotion has also been observed in other anatomical regions of hominoid primates, such as the insertion sites of the ligaments holding the flexor digitorum profundus and superficialis muscles in proximal phalanges [39]. In addition to this association between morphological characteristics and type of locomotion, a further reason to analyze the RSC + LRL and the SRL insertion sites is that the landmarks are easily placed, which helps eliminate the possibility of intra- or inter-observer error. Finally, the role of the palmar ligaments as the main stabilizing elements of the wrist [37] makes them crucial to understanding the function of the wrist in primates.

In the present study, we have analyzed by tridimensional geometric morphometrics (3D GM) the morphology of the insertion site of the palmar radiocarpal ligaments in these three species of hominoid primates (*Gorilla gorilla, Pan troglodytes*, and *Pongo pygmaeus*), comparing wild and captive individuals. Our main objectives were to quantify the distal radius of hominoid primates to see if morphological differences could be observed between wild and captive subjects. Since the anatomy of the insertion sites of the RSC, LRL, and SRL is related to type of locomotion, we hypothesized that quantifiable morphological differences would exist in the ligament insertion sites of the distal radial epiphysis between wild and captive individuals of the three species as a result of their different types of locomotion [2,7,16]. Specifically, we expected that the morphology of the ligament insertion sites in captive chimpanzees, which tend to use terrestrial locomotion [2], would be more similar to that of gorillas than that of wild chimpanzees. In contrast, the morphology of the ligament insertion sites in captive orangutans and gorillas, which prefer to stay on elevated structures [12,16,17,26], will translate the greater load in the wrist region. Our findings would increase current knowledge of how captive conditions can influence the locomotor pattern of hominoid primates and modify the anatomical characteristics of upper-limb joint complexes like the wrist. We also believe that follow-up studies are warranted to determine how our results may be applied to improve the enclosures and environments of primates in captivity and make them as similar as possible to the environments found in the wild.

## 2. Materials and Methods

### 2.1. Osteological Samples

A total of 118 left radii were included in the study: 51 from *Pan troglodytes* (25 wild and 26 captive); 43 from *Gorilla gorilla* (31 wild and 12 captive); and 24 from *Pongo pygmaeus* (15 wild and 9 captive) (Table 1). All the radii used in the present analysis belong to primates that died from causes unrelated to the present study. The primates raised in the wild were provided by the Anthropologisches Institut und Museum (University of Zurich, Zürich, Switzerland), and the primates bred in captivity, by the Museu de Ciències Naturals de Barcelona (Barcelona, Spain) and the Museo Anatómico de la Universidad de Valladolid (Valladolid, Spain). All specimens were from adult primates, as defined by the presence of fused epiphyses and the third molar. The captive primates used in this study came from various Spanish zoological parks (Madrid Zoo, Barcelona Zoo, Loro Parque de Tenerife, Bioparc de Valencia, Zoo de Fuengirola, Zoo Valwo de Valladolid, and Zoo de Santillana del Mar). The wild chimpanzees and gorillas analyzed came from Equatorial Guinea, Gabon, Liberia, and Cameroon, while the orangutans came from Borneo and Sumatra.

### 2.2. 3D GM Analysis

The distal radial epiphyses were scanned with a 3D Next Engine Ultra HD laser surface scanner at a resolution of 0.1 mm space-point separation, with a density of 40 k (2×) points. The different sections of the scans were merged with the Volume Merge option of Next Engine HD software at a resolution of 0.5 mm and saved as a PLY file. The resulting triangle mesh was edited with the open-source MeshLab software [40], and the models were imported into Landmark Editor software (v. 3.6) [41] for placing the landmarks.

The landmarks previously proposed by Casado et al. [38] were used to represent the morphology of the two insertion sites of the palmar radiocarpal ligaments in the distal radial epiphysis. Nine Type II and one Type III landmarks were used: the L1–L4 landmarks for the SRL insertion site and the L5–L10 landmarks for the common insertion site of the RSC and LRL (Figure 1). The raw data obtained with Landmark Editor software based on the landmark coordinates were exported into the MorphoJ statistical package [42].

In order to confirm the reliability of the landmarks, we established two protocols to calculate intra-observer error and inter-observer error before beginning the analyses. For intra-observer error, each observer placed all the landmarks in the sample on three separate days, with a 48 h interval between the days. Inter-observer error was calculated at the same time as intra-observer error. Two additional experienced observers placed all the landmarks in the sample on three separate days, with a 48 h interval between the days. The 48 h interval was to rule out the possibility of an observer placing the landmarks through a mechanical repetition of previous placements. Differences were analyzed with a pairwise Mann–Whitney analysis in order to detect any lack of reliability in the landmarks or the data.

A generalized Procrustes analysis (GPA) was used to reduce variability due to differences of size, placement, or orientation and to minimize the sum of square distances between equivalent landmarks [42,43,44,45]. This procedure allows the resulting data, termed Procrustes coordinates, to be used in a multivariate analysis [44]. A principal components analysis (PCA) was then performed in order to reduce complex multidimensional data to fewer components, or eigenvectors, which could be used to explain the main differences between the groups [42,43,44,45].

After the PCA, sample normality was tested in PAST software using the Shapiro–Wilk and Anderson–Darling tests. Variation in species was tested with a Procrustes ANOVA with permutation, including status (wild–captive). In order to determine the potential effect of sex on the morphological characteristics of the ligament insertion sites, we performed a discriminant function analysis (DFA) for all groups and other DFA for each of the species, controlling for sex and status. The groups were classified using a DFA with Fisher’s classification rule and leave-one-out cross-validation. Subsequently, we performed a MANCOVA with species as group and centroid size as covariate [44] for each of the species, using log-transformed centroid size to increase the accuracy of the model.

In order to determine the influence of size on variation in shape (allometric scaling), a multivariate regression analysis (MRA) with a permutation test with 1000 randomizations was performed. Procrustes coordinates, indicative of shape, was the dependent variable, and the centroid size (CS), indicative of size, was the independent variable [42,43,44,45]. After corroborating the allometric influence of the sample, a second PCA was performed with the regression residuals.

## 3. Results

The analysis of intra-observer error and inter-observer error revealed no significant differences (Supplementary Table S1).

**Figure 2:**
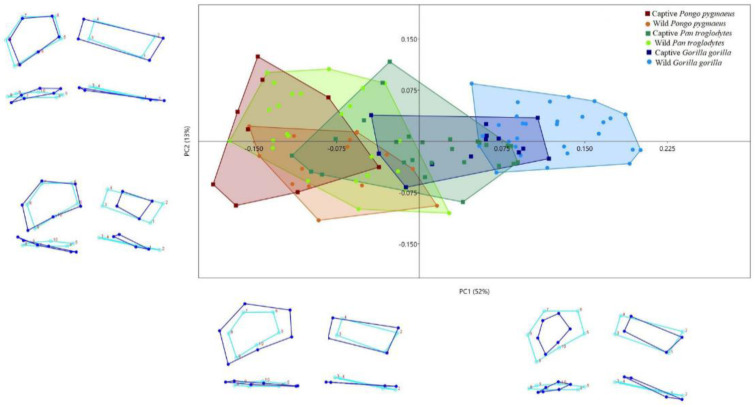
Convex Hull of PC1 vs. PC2 derived from the PCA of the 3D GM analysis. Dark blue wireframes show the extreme shape of each PC in a palmar view (upper panel) and a proximal view (lower panel). Light blue wireframes show the mean shape (coordinates 0.0).

The PCA yielded 23 PCs, the first three of which accounted for 72.4% of the variation in the shape of the two insertion sites of the palmar radiocarpal ligaments (PC1, 52.5%; PC2, 12.7%; PC3, 7.2%). The remaining PCs accounted for ≤5% each of the variation in shape. The scatterplot of PC1 vs. PC2 (Figure 2) shows differences among the six groups of primates, although there is a clear degree of overlap. The wild and captive *Pongo pygmaeus* and the wild *Pan troglodytes* were mainly located in the negative PC1 values, while the wild and captive *Gorilla gorilla* were mainly located in the positive values, and the captive *Pan troglodytes* were mainly located in an intermediate position between the positive and negative values. More positive PC1 values could be seen in wild than in captive *Gorilla gorilla* and *Pongo pygmaeus*, while more positive PC1 values were seen in captive than in wild *Pan troglodytes*. Specimens with positive PC1 values were characterized by relatively small insertion sites of the palmar radiocarpal ligaments and by an ulnopalmar orientation of the SRL insertion site in relation to the RSC + LRL insertion site. In contrast, in specimens with negative PC1 values, the two insertion sites were relatively large, and the SRL insertion site had a palmar orientation in relation to the RSC + LRL insertion site.

In contrast, more positive PC2 values were seen in captive *Pongo pygmaeus*, while more negative values were seen in wild *Pongo pygmaeus*. Specimens with positive PC2 values had a relatively large and palmarly oriented insertion site of the SRL in relation to the RSC + LRL insertion site, while specimens with negative PC2 values had a relatively small and ulnopalmarly oriented insertion site of the SRL in relation to the RSC + LRL insertion site (Figure 2).

Procrustes ANOVA showed significant differences between wild and captive specimens in centroid size (SS = 5601.35, MS = 2800.67, df = 2, F = 45.56, *p* = 0.005) and in shape (SS = 0.74, MS = 0.02, df = 46, F = 4.8, *p* < 0.001). The DFA of the effect of sex showed no significant differences between males and females in the morphology of the ligament insertion sites, either in Procrustes distances (Pd = 0.02, *p* = 0.26) or in Mahalanobis distances (Md = 0.94, *p* = 0.56). However, when status was included in the analysis, significant differences were observed among captive gorillas in Procrustes distances (Pd = 0.08, *p* = 0.03) but not in Mahalanobis distances (Md = 3.58, *p* = 0.88). There were no significant differences according to sex among wild gorillas, (Pd = 0.02, *p* = 0.71; Md = 3.83, *p* = 0.55), captive chimpanzees (Pd = 0.03, *p* = 0.48; Md = 10.51, *p* = 0.31), wild chimpanzees (Pd = 0.03, *p* = 0.91; Md = 2.91, *p* = 0.96), captive orangutans (Pd = 0.10, *p* = 0.40; Md = 1.44, *p* = 0.40), or wild orangutans (Pd = 0.04, *p* = 0.84; Md = 3.35, *p* = 0.25). The DFA showed significant differences in Procrustes distances between wild and captive specimens of all three species. It also showed significant differences in Mahalanobis distances for wild vs. captive gorillas and chimpanzees but not for orangutans (Table 2). Leave-one-out cross validations showed that the post hoc probabilities of correct classification decreased in all the comparisons (Table 3).

**Figure 3:**
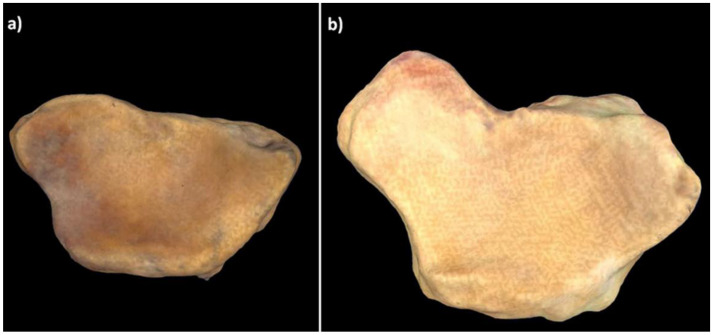
Distal view of the carpal articular surface of radius in (**a**) a gorilla with low allometric effect and (**b**) a gorilla with great allometric effect. A marked displacement of the palmar margin of the lunate fossa can be observed in (**b**).

The MANCOVA showed a predictive value of 16.49% (Tot SS = 0.13, Pred SS = 0.02, Res SS = 0.10, *p* = 0.04) for captive gorillas, 11.68% (Tot SS = 0.20, Pred SS = 0.02, Res SS = 0.18, *p* = 0.002) for wild gorillas, 4.82% (Tot SS = 0.27, Pred SS = 0.01, Res SS = 0.26, *p* = 0.258) for captive chimpanzees, 4.56% (Tot SS = 0.31, Pred SS = 0.01, Res SS = 0.30, *p* = 0.33) for wild chimpanzees, 7.56% (Tot SS = 0.13, Pred SS = 0.01, Res SS = 0.12, *p* = 0.776) for captive orangutans, and 15.75% (Tot SS = 0.17, Pred SS =0.03, Res SS = 0.14, *p* = 0.009) for wild orangutans. Both wild and captive gorillas with allometric effect showed an axial rotation of the RSC + LRL and SRL insertion sites associated with a displacement of the palmar margin of the lunate fossa (Figure 3). Wild orangutans with significant MANCOVA values had a larger depression in the RSC + LRL insertion site.

**Figure 4:**
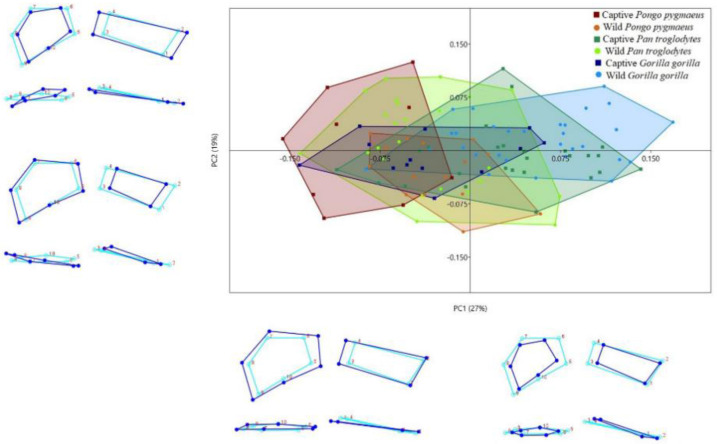
Convex Hull of PC1 vs. PC2 derived from the PCA post regression of the 3D GM analysis. Dark blue wireframes show the extreme shape of each PC in a palmar view (upper panel) and a proximal view (lower panel). Light blue wireframes represent the mean shape (coordinates 0.0).

The MRA of shape over CS found that 27.12% of the variation in the shape of the insertion sites of the palmar radiocarpal ligaments was attributable to size (*p* < 0.001). The second PCA, performed with the MRA residuals, yielded 23 PCs, the first 6 of which accounted for 76% of the total variation in the shape of the insertion sites of the palmar radiocarpal ligaments (PC1, 27.5%; PC2, 19.5%; PC3, 8.9%; PC4, 7.6%; PC5, 6.6%; PC6, 5.9%). The remaining PCs accounted for <5% each of the variation in shape. The scatterplot of PC1 vs. PC2 (Figure 4) shows that wild *Gorilla gorilla* and captive *Pan troglodytes* are mainly located in the positive PC1 values, while the other four types of specimens are mainly located in the negative PC1 values. More positive PC1 values could be seen in wild than in captive *Gorilla gorilla* and *Pongo pygmaeus*, while more positive PC1 values were seen in captive than in wild *Pan troglodytes*. The shape changes observed in PC1 in the second PCA were similar to those observed with the first PCA using the Procrustes coordinates. More positive PC2 values were seen in captive *Pongo pygmaeus*, while more negative values were seen in wild *Pongo pygmaeus*. Among specimens with positive PC2 values, the SRL insertion site was relatively large with a palmar orientation in relation to the RSC + LRL insertion site, while the RSC + LRL insertion site was relatively small. Furthermore, in specimens with negative PC2 values, the SRL insertion site was relatively small, with an ulnopalmar orientation in relation to the RSC + LRL insertion site, while the RSC + LRL insertion site was relatively large (Figure 4).

## 4. Discussion

We have analyzed by 3D GM the insertion sites of the palmar radiocarpal ligaments in specimens from *Gorilla gorilla*, *Pan troglodytes*, and *Pongo pygmaeus*. Our findings indicate that there are morphological differences both between species and between wild and captive individuals of the same species that may be a result of differences in locomotor behavior. Moreover, these differences were not affected by the sex of the specimen, except in captive gorillas, where there were differences between males and females in Procrustes but not in Mahalanobis distances. In the first PCA (Figure 2), the distribution of the three species in PC1 was similar to that previously described by our team [38]: positive PC1 values for gorillas, negative values for orangutans, and intermediate values for chimpanzees. Gorillas are the least arboreal of the three species and use knuckle-walking more frequently than the other two [27,45], which can explain their relatively small ligament insertion sites. The increased stability of the scaphoid and lunate bones, an adaptation to knuckle-walking [46], reduces the need for large ligaments to stabilize the radiocarpal joint. In contrast, orangutans are the most arboreal of the three species [47] and have relatively large ligament insertion sites. Their palmar radiocarpal ligaments are more highly developed to compensate for the large traction forces affecting the wrist during vertical climbing [48,49]. Orangutans are also characterized by a relative palmar orientation of the SRL insertion site, which is likely a result of the larger size of the radiolunate joint [50], which allows for the greater loading of the joint that occurs during vertical climbing [48,51]. Finally, chimpanzees, in an intermediate position between gorillas and orangutans, use a more varied locomotion, combining knuckle-walking with arboreal locomotion [52,53,54]. When the effect of size on shape variation (allometric scaling) (Figure 4) was eliminated, the most significant change was the displacement of captive gorillas toward negative PC1 values and of captive chimpanzees toward positive PC1 values. Nonetheless, there were still significant differences between wild and captive specimens of each of the three species, indicating that conditions of captivity can lead to changes in the development of locomotor behavior of hominoid primates that can modify the anatomical characteristics of large joint complexes like the wrist [2].

Both PCAs showed that wild gorillas had more positive PC1 values than captive gorillas, indicating that the insertion sites of the palmar radiocarpal ligaments are relatively larger in captive than in wild gorillas (Figure 2 and Figure 4). Since a small size of these insertion sites is related to knuckle-walking and a larger size to suspensory behavior or vertical climbing [38], we can infer that captive gorillas climb from the ground to the elevated places of their habitat more frequently than do wild gorillas. Although few studies have been conducted comparing the locomotion of wild vs. captive gorillas, some have shown that captive gorillas have a greater preference for elevated places in their habitats, such as rocks or trees, than for the flat places of the substrate [26,27]. Furthermore, the locomotor behavior of captive gorillas is more often limited to vertical substrates, because the artificial material of these substrates is less prone to breakage than the branches of natural trees, making them easily usable by large gorillas [27]. Although gorillas are the least arboreal of all hominoid primates [46], they are capable of using different types of arboreal locomotion, especially vertical climbing, to seek food or to build nests [55], and captive gorillas tend to build their nests in elevated places more often than their wild counterparts [56]. Interestingly, the analysis of the cross-section of the humerus diaphysis by Canington et al. [9] revealed significant differences between wild and captive gorillas. In fact, the adult specimens of wild *Gorilla gorilla* were more like adult specimens of *Gorilla beringei*, which is more terrestrial than *Gorilla gorilla*. This is in line with our findings that the morphology of the insertion sites of the palmar radiocarpal ligaments is more typical of knuckle-walkers in wild than in captive gorillas (Figure 2 and Figure 4).

Unlike gorillas, the captive chimpanzees had more positive PC1 values than their wild counterparts (Figure 2 and Figure 4). The relatively small size of the insertion sites of the palmar radiocarpal ligaments in captive chimpanzees could indicate that they rely on knuckle-walking more often than wild chimpanzees. Chimpanzees are known to be less terrestrial than gorillas [46] and are estimated to spend 33.68% of their time in trees when living in the wild [12]. This arboreal behavior is more evident in infantile and juvenile individuals, whose behavior is generally suspensory, dominated by the upper extremity, while adults exhibit more terrestrial locomotion, especially knuckle-walking [53,57,58]. Captive chimpanzees also have a preference for elevated sites in their habitats [13,15,27] and are estimated to spend up to 40% of their time in structures above ground level [59]. Despite this preference of wild and captive chimpanzees for elevated substrates, captive juvenile chimpanzees use knuckle-walking more frequently than do wild chimpanzees [2,60]. The higher frequency of knuckle-walking in captive juvenile chimpanzees could explain the differences in the morphology of the insertion sites of palmar radiocarpal ligaments between our wild and captive chimpanzees. Osteoarticular structures in developing individuals are more susceptible to differences in loading [61], and the captive juveniles use knuckle-walking more frequently, while wild juveniles use arboreal locomotion more than adult specimens [58]. The greater amount of time that captive juveniles spend on the ground, in many cases knuckle-walking on relatively rigid surfaces, also explains other anatomical differences that have been observed in the wrist region between wild and captive chimpanzees, such as the larger relative size of the surface of the distal radial epiphysis in captive specimens [2]. An additional factor may explain the reduced use of arboreal locomotion in captive chimpanzees: wild chimpanzees spend up to 50% of their time searching for food in trees [62], while captive chimpanzees spend less time on this activity [63], even in large naturalistic enclosures [64].

Wild orangutans had more positive PC1 values than captive orangutans, indicating that the insertion sites of the palmar radiocarpal ligaments are relatively large in captive specimens (Figure 2 and Figure 4). The few studies that have analyzed differences in locomotion between wild and captive orangutans have found that both wild and captive individuals prefer elevated places. They avoid moving along the ground and spend up to two-thirds of their time in trees or on elevated platforms [16]. Still, the differences we have observed in the ligament insertion sites can be explained by the mechanical differences in vertical climbing between wild and captive orangutans. Vertical climbing accounts for 22–26% of the total locomotion time of orangutans—in adults and juveniles and in males and females [7,65]. Wild orangutans use a more cautious, slow, short-step vertical climbing because of their complex habitat ecology [66], while captive orangutans are less cautious and faster with longer steps, possibly because, in captivity, the individuals are more familiar with the fixed and stable structures used for climbing their artificial habitat [7]. This faster vertical climbing with longer steps can put extra load on the wrist, which would explain the larger size of the palmar radiocarpal ligaments in the captive orangutans, necessary to ensure the stability of the radiocarpal joint [38,49,50]. Wild orangutans prefer to climb using vines or narrow tree trunks with flexed-elbow vertical climbing [67], while captive orangutans more frequently use extended-elbow vertical climbing, which involves a longer support phase and is, hence, more mechanically demanding [7]. This increased mechanical requirement entails a greater development of the palmar radiocarpal ligaments, resulting in relatively large insertion sites (Figure 2 and Figure 4), particularly for the SRL, as evidenced by the PC2 values in our study. The SRL is an important stabilizer of the radiolunate joint during vertical climbing [49,52], and the large size and palmar orientation of its insertion site in captive orangutans compared to their wild counterparts helps to compensate for the increased mechanical demands required by extended-elbow vertical climbing.

In addition to these morphological differences between wild and captive individuals, our MANCOVA identified allometric scaling effects in wild and captive gorillas and in wild orangutans. Both the wild and captive gorillas with a greater allometric effect had an axial rotation that affected both the RSC + LRL and the SRL insertion sites. This axial rotation was associated with expansion and concavation of the carpal articular surface of radius, which could be due to the palmar–distal displacement of the palmar margin of the lunate fossa, as shown in the 3D models (Figure 3). The wild orangutans with a greater allometric effect had a depressed RSC + LRL insertion site, which was also seen in the 3D models.

## 5. Conclusions

In conclusion, our 3D GM analysis has identified morphological differences in the insertion sites of the palmar radiocarpal ligaments between wild and captive orangutans, chimpanzees, and gorillas. These differences suggest that conditions of captivity can lead to changes in the locomotor behavior of hominoid primates that can modify the anatomy of some regions of the postcranial skeleton, such as the wrist. Our results support the idea advocated by some authors that bones from captive primates should be used cautiously in osteological studies, as the functional implications of these studies may be highly conditioned by the conditions of captivity [1,3]. This is particularly evident in the distal radial epiphysis, where other anatomical differences between wild and captive hominoid primates [2] have also been identified. We hope that our results can help to improve the habitats of captive hominoid primates by enriching the spaces and creating similar dynamics to their natural wild environment [64,68]. In this way, captive specimens could recreate as closely as possible the locomotor behaviors that they would develop in natural environments. In addition, our findings can be useful in studies of comparative anatomy. For example, the fact that captive conditions can modify the morphology of muscle and ligament insertion sites leads us to suggest that future studies reconstructing the locomotor behavior patterns of fossil primates based on a comparison with bone specimens of present-day primates should, whenever possible, use bones of individuals born and raised in the wild.

## 6. Limitations and Future Directions

The main limitation of our study is the relatively limited number of specimens analyzed, especially *Pongo pygmaeus*. For this reason, we believe that future studies should include more specimens to further explore the morphological differences observed in the present study and determine if they are truly representative. In addition, since we analyzed only one specific anatomical region, in order to determine more precisely how different locomotor behaviors can modify bone morphology in captive primates, we suggest including other regions, such as the elbow, the shoulder, the knee, and the hip. The methods used in the present study could also be used to see if these differences occur equally in the right and left side of these primates. Furthermore, we consider that, in future studies, it would be interesting to study the body mass of captive primates in situ to corroborate if their body mass is directly related to bone dysmorphologies. Finally, our group is currently exploring the specific locomotor behaviors developed by captive primates with the aim of identifying common patterns related to the morphological changes we have observed in this study.

## Figures and Tables

**Table 1:** Radius specimens used for the 3D GM analysis. M = Male, F = Female, I = Indeterminate.

Species	*n*	Sex	Origin
*Gorilla gorilla* (Wild)	31	M = 17/F = 14	Equatorial Guinea, Cameroon, Gabon, French Cameroon
*Gorilla gorilla* (Captive)	12	M = 6/F = 6	Madrid Zoo, Loro Parque de Tenerife, Fuengirola Zoo, Bioparc de Valencia, Barcelona Zoo
*Pan troglodytes* (Wild)	25	M = 11/F = 13/I = 1	Equatorial Guinea, Liberia, French Cameroon
*Pan troglodytes* (Captive)	26	M = 15/F = 11	Valladolid Valwo Zoo, Fuengirola Zoo, Madrid Zoo, Barcelona Zoo
*Pongo pygmaeus* (Wild)	15	M = 8/F = 7	Sumatra, Borneo
*Pongo pygmaeus* (Captive)	9	M = 2/F = 7	Santillana del Mar Zoo, Fuengirola Zoo, Madrid Zoo, Barcelona Zoo
TOTAL	118		Wild = 71 Captive= 47

**Table 2:** Procrustes and Mahalanobis distances between wild and captive specimens.

Species	Procrustes Distances	Mahalanobis Distances
Wild vs. Captive *Gorilla gorilla*	0.09 (*p* < 0.0001)	4.03 (*p* = 0.01)
Wild vs. Captive *Pan troglodytes*	0.08 (*p* < 0.0001)	2.70 (*p* = 0.01)
Wild vs. Captive *Pongo pygmaeus*	0.08 (*p* = 0.006)	33.93 (*p* = 0.07)

**Table 3:** Percentages of correct post hoc classification from the discriminant functions and after leave-one-out cross-validation, with the percentage of decrease in correct classification.

	Discriminant Functions	After Cross-Validation	Decrease in Correct Classification
Wild vs. Captive *Gorilla gorilla*	96.66%	63.58%	33.08%
Wild vs. Captive *Pan troglodytes*	94.16%	64.69%	29.47%
Wild vs. Captive *Pongo pygmaeus*	100%	69.99%	30.01%

## Data Availability

We have included details on the samples used in the study on figshare (https://figshare.com/projects/Wild_vs._Captive/97894) Casado Rodríguez, Aroa (22 February 2021): Figure 1. figshare. Figure. https://doi.org/10.6084/m9.figshare.13714141.v5 Casado Rodríguez, Aroa (27 April 2021): LBVS.CV. figshare. Dataset. https://doi.org/10.6084/m9.figshare.13713907.v3 Casado Rodríguez, Aroa (27 April 2021): Dataset Wildvs.Captivity. figshare. Dataset. https://doi.org/10.6084/m9.figshare.13714099.v4 Casado Rodríguez, Aroa (27 April 2021): Figure 2. figshare. Figure. https://doi.org/10.6084/m9.figshare.13714159.v3 Casado Rodríguez, Aroa (27 April 2021): Figure 3. figshare. Figure https://doi.org/10.6084/m9.figshare.14077421.v2 Casado Rodríguez, Aroa (27 April 2021): Figure 4. figshare. Figure. https://doi.org/10.6084/m9.figshare.14077424.v2 Casado Rodríguez, Aroa (27 April 2021). We are unable to share data on the 3D models, because these data are owned by a third-party organization. For information on these data, please contact Anthropologisches Institut und Museum of the University of Zurich (aim-collection@ifi.uzh.ch), Museo Anatómico de la Universidad de Valladolid (juanpas@med.uva.es), and Museo de Ciencias Naturales de Barcelona (jquesada@bcn.cat).

## Supplementary Materials

The following are available online at https://www.mdpi.com/article/10.3390/ani11071856/s1, Table S1: Results of the pairwise Mann-Whitney analysis of intra-and inter-observer error. The first number in the heading indicates the number of the observer (observer 1, 2, or 3) and the final number indicates the day the analysis was performed (day 1, 2, or 3).
