# Supplementary material for: Effects of Captivity on the Morphology of the Insertion Sites of the Palmar Radiocarpal Ligaments in Hominoid Primates
Source: Animals (Basel). 2021 Jun 22;11(7):1856. doi: 10.3390/ani11071856 (PMC8300253; doi:10.3390/ani11071856)
Supplement: Supplementary file 1 [file animals-11-01856-s001.zip › animals-1232588-supplementary.pdf]

Supplementary Table S1. Results of the pairwise Mann-Whitney analysis of intra- and inter-observer error. The first number in the heading indicates the number of the observer (observer 1, 2, or 3) and the final number indicates the day the analysis was performed (day 1, 2, or 3).

	1/PC1/1	1/PC2/1	1/PC3/1	1/PC1/2	1/PC2/2	1/PC3/2	1/PC1/3	1/PC2/3	1/PC3/3
**1/PC1/1**		0.9855	0.9445	0.9992	0.9855	0.9445	0.9962	0.9992	0.9506
**1/PC2/1**	0.9855		0.9855	0.9855	0.9992	0.9855	0.9112	0.981	0.9082
**1/PC3/1**	0.9445	0.9855		0.9445	0.9855	0.9992	0.89	0.9688	0.9384
**1/PC1/2**	0.9992	0.9855	0.9445		0.9855	0.9445	0.9962	0.9992	0.9506
**1/PC2/2**	0.9855	0.9992	0.9855	0.9855		0.9855	0.9112	0.981	0.9082
**1/PC3/2**	0.9445	0.9855	0.9992	0.9445	0.9855		0.89	0.9688	0.9384
**1/PC1/3**	0.9962	0.9112	0.89	0.9962	0.9112	0.89		0.8705	0.9142
**1/PC2/3**	0.9992	0.981	0.9688	0.9992	0.981	0.9688	0.8705		0.8915
**1/PC3/3**	0.9506	0.9082	0.9384	0.9506	0.9082	0.9384	0.9142	0.8915	
**2/PC1/1**	0.984	0.7494	0.7393	0.984	0.7494	0.7393	0.9992	0.7814	0.7756
**2/PC2/1**	0.9962	0.9673	0.9203	0.9962	0.9673	0.9203	0.8615	0.9795	0.8976
**2/PC3/1**	0.9855	0.872	0.7873	0.9855	0.872	0.7873	0.9278	0.8585	0.6895
**2/PC1/2**	0.9734	0.8212	0.7844	0.9734	0.8212	0.7844	0.9992	0.7991	0.7683
**2/PC2/2**	0.9992	0.9688	0.9992	0.9992	0.9688	0.9992	0.8735	0.9734	0.984
**2/PC3/2**	0.943	0.9339	0.946	0.943	0.9339	0.946	0.8735	0.9445	0.946
**2/PC1/3**	0.9992	0.9491	0.9734	0.9992	0.9491	0.9734	0.9779	0.981	0.9688
**2/PC2/3**	0.9901	0.9795	0.9127	0.9901	0.9795	0.9127	0.9172	0.9992	0.851
**2/PC3/3**	0.9719	0.9354	0.8227	0.9719	0.9354	0.8227	0.8885	0.9036	0.7814
**3/PC1/1**	0.9764	0.9871	0.9475	0.9764	0.9871	0.9475	0.9475	0.9977	0.9491
**3/PC2/1**	0.9825	0.9324	0.9673	0.9825	0.9324	0.9673	0.8931	0.9536	0.981
**3/PC3/1**	0.9703	0.9977	0.9354	0.9703	0.9977	0.9354	0.9127	0.9962	0.9673
**3/PC1/2**	0.9551	0.9218	0.9021	0.9551	0.9218	0.9021	0.9779	0.89	0.9157
**3/PC2/2**	0.9901	0.8946	0.9749	0.9901	0.8946	0.9749	0.9006	0.9369	0.9521
**3/PC3/2**	0.9749	0.9855	0.8675	0.9749	0.9855	0.8675	0.9369	0.9536	0.8064
**3/PC1/3**	0.9855	0.8615	0.863	0.9855	0.8615	0.863	0.9612	0.851	0.842
**3/PC2/3**	0.9916	0.8585	0.8931	0.9916	0.8585	0.8931	0.9172	0.8465	0.9248
**3/PC3/3**	0.9734	0.9384	0.9764	0.9734	0.9384	0.9764	0.8946	0.9932	0.9354

3/PC3/1	3/PC2/1	3/PC1/1	2/PC3/3	2/PC2/3	2/PC1/3	2/PC3/2	2/PC2/2	2/PC1/2	2/PC3/1	2/PC2/1	2/PC1/1
0.9703	0.9825	0.9764	0.9719	0.9901	0.9992	0.943	0.9992	0.9734	0.9855	0.9962	0.984
0.9977	0.9324	0.9871	0.9354	0.9795	0.9491	0.9339	0.9688	0.8212	0.872	0.9673	0.7494
0.9354	0.9673	0.9475	0.8227	0.9127	0.9734	0.946	0.9992	0.7844	0.7873	0.9203	0.7393
0.9703	0.9825	0.9764	0.9719	0.9901	0.9992	0.943	0.9992	0.9734	0.9855	0.9962	0.984
0.9977	0.9324	0.9871	0.9354	0.9795	0.9491	0.9339	0.9688	0.8212	0.872	0.9673	0.7494
0.9354	0.9673	0.9475	0.8227	0.9127	0.9734	0.946	0.9992	0.7844	0.7873	0.9203	0.7393
0.9127	0.8931	0.9475	0.8885	0.9172	0.9779	0.8735	0.8735	0.9992	0.9278	0.8615	0.9992
0.9962	0.9536	0.9977	0.9036	0.9992	0.981	0.9445	0.9734	0.7991	0.8585	0.9795	0.7814
0.9673	0.981	0.9491	0.7814	0.851	0.9688	0.946	0.984	0.7683	0.6895	0.8976	0.7756
0.7523	0.78	0.9658	0.7221	0.725	0.9309	0.7509	0.7697	0.9871	0.7065	0.761	
0.9779	0.8885	0.9612	0.9551	0.9901	0.9886	0.9324	0.9932	0.8109	0.9688		0.761
0.8495	0.7858	0.9779	0.9354	0.8735	0.9688	0.7193	0.7991	0.7683		0.9688	0.7065
0.78	0.842	0.94	0.7581	0.7697	0.9218	0.8064	0.8123		0.7683	0.8109	0.9871
0.9354	0.984	0.9916	0.8525	0.9567	0.9643	0.9734		0.8123	0.7991	0.9932	0.7697
0.9369	0.9627	0.8946	0.7873	0.8555	0.984		0.9734	0.8064	0.7193	0.9324	0.7509
0.9779	0.9734	0.9612	0.9992	0.9536		0.984	0.9643	0.9218	0.9688	0.9886	0.9309
0.9521	0.9036	0.9932	0.9218		0.9536	0.8555	0.9567	0.7697	0.8735	0.9901	0.725
0.8765	0.8765	0.9293		0.9218	0.9992	0.7873	0.8525	0.7581	0.9354	0.9551	0.7221
0.9795	0.9734		0.9293	0.9932	0.9612	0.8946	0.9916	0.94	0.9779	0.9612	0.9658
0.9066		0.9734	0.8765	0.9036	0.9734	0.9627	0.984	0.842	0.7858	0.8885	0.78
	0.9066	0.9795	0.8765	0.9521	0.9779	0.9369	0.9354	0.78	0.8495	0.9779	0.7523
0.9066	0.9218	0.9673	0.8645	0.9112	0.981	0.9203	0.8991	0.9369	0.9021	0.9293	0.9112
0.8615	0.946	0.9855	0.7946	0.8825	0.9506	0.9582	0.9354	0.7946	0.7393	0.9324	0.7639
0.9172	0.8257	0.9597	0.9627	0.878	0.9977	0.8406	0.8495	0.7917	0.9066	0.984	0.7221
0.854	0.9006	0.9491	0.8346	0.878	0.9673	0.875	0.8376	0.9703	0.869	0.872	0.9992
0.8079	0.8946	0.9855	0.761	0.8645	0.9947	0.8765	0.8331	0.8361	0.6811	0.845	0.7858
0.9506	0.943	0.9415	0.845	0.9082	0.9764	0.981	0.9962	0.7932	0.7581	0.9248	0.7581

3/PC3/3	3/PC2/3	3/PC1/3	3/PC3/2	3/PC2/2	3/PC1/2
0.9734	0.9916	0.9855	0.9749	0.9901	0.9551
0.9384	0.8585	0.8615	0.9855	0.8946	0.9218
0.9764	0.8931	0.863	0.8675	0.9749	0.9021
0.9734	0.9916	0.9855	0.9749	0.9901	0.9551
0.9384	0.8585	0.8615	0.9855	0.8946	0.9218
0.9764	0.8931	0.863	0.8675	0.9749	0.9021
0.8946	0.9172	0.9612	0.9369	0.9006	0.9779
0.9932	0.8465	0.851	0.9536	0.9369	0.89
0.9354	0.9248	0.842	0.8064	0.9521	0.9157
0.7581	0.7858	0.9992	0.7221	0.7639	0.9112
0.9248	0.845	0.872	0.984	0.9324	0.9293
0.7581	0.6811	0.869	0.9066	0.7393	0.9021
0.7932	0.8361	0.9703	0.7917	0.7946	0.9369
0.9962	0.8331	0.8376	0.8495	0.9354	0.8991
0.981	0.8765	0.875	0.8406	0.9582	0.9203
0.9764	0.9947	0.9673	0.9977	0.9506	0.981
0.9082	0.8645	0.878	0.878	0.8825	0.9112
0.845	0.761	0.8346	0.9627	0.7946	0.8645
0.9415	0.9855	0.9491	0.9597	0.9855	0.9673
0.943	0.8946	0.9006	0.8257	0.946	0.9218
0.9506	0.8079	0.854	0.9172	0.8615	0.9066
0.9112	0.8976	0.9779	0.9203	0.9278	
0.9339	0.9597	0.8946	0.7465		0.9278
0.845	0.6783	0.8495		0.7465	0.9203
0.875	0.9278		0.8495	0.8946	0.9779
0.872		0.9278	0.6783	0.9597	0.8976
	0.872	0.875	0.845	0.9339	0.9112
